# Rag1 immunodeficiency‐induced early aging and senescence in zebrafish are dependent on chronic inflammation and oxidative stress

**DOI:** 10.1111/acel.13020

**Published:** 2019-07-26

**Authors:** Beatriz Novoa, Patricia Pereiro, Azucena López‐Muñoz, Mónica Varela, Gabriel Forn‐Cuní, Monique Anchelin, Sonia Dios, Alejandro Romero, Alicia Martinez‐López, Regla María Medina‐Gali, Manuel Collado, Julio Coll, Amparo Estepa, María Luisa Cayuela, Victoriano Mulero, Antonio Figueras

**Affiliations:** ^1^ Instituto de Investigaciones Marinas Consejo Superior de Investigaciones Científicas (CSIC) Vigo Spain; ^2^ Departamento de Biología Celular e Histología, Facultad de Biología, Universidad de Murcia IMIB‐Arrixaca Murcia Spain; ^3^ Grupo de Telomerasa, Cáncer y Envejecimiento, Hospital Clínico Universitario Virgen de la Arrixaca IMIB‐Arrixaca Murcia Spain; ^4^ Instituto de Biología Molecular y Celular (IBMC) Universidad Miguel Hernández (UMH) Elche Spain; ^5^ Instituto de Investigación Sanitaria de Santiago de Compostela (IDIS), Complexo Hospitalario Universitario de Santiago de Compostela (CHUS) SERGAS Santiago de Compostela Spain; ^6^ Departamento de Biotecnología Instituto Nacional Investigación y Tecnología Agraria y Alimentaria (INIA) Madrid Spain

**Keywords:** aging, DNA damage, inflammation, oxidative stress, *rag1*, senescence

## Abstract

In mammals, recombination activating gene 1 (RAG1) plays a crucial role in adaptive immunity, generating a vast range of immunoglobulins. *Rag1* ^−/−^ zebrafish (*Danio rerio*) are viable and reach adulthood without obvious signs of infectious disease in standard nonsterile conditions, suggesting that innate immunity could be enhanced to compensate for the lack of adaptive immunity. By using microarray analysis, we confirmed that the expression of immunity‐ and apoptosis‐related genes was increased in the *rag1* ^−/−^ fish. This tool also allows us to notice alterations of the DNA repair and cell cycle mechanisms in *rag1* ^−/−^ zebrafish. Several senescence and aging markers were analyzed. In addition to the lower lifespan of *rag1* ^−/−^ zebrafish compared to their wild‐type (wt) siblings, *rag1* ^−/−^ showed a higher incidence of cell cycle arrest and apoptosis, a greater amount of phosphorylated histone H2AX, oxidative stress and decline of the antioxidant mechanisms, an upregulated expression and activity of senescence‐related genes and senescence‐associated β‐galactosidase, respectively, diminished telomere length, and abnormal self‐renewal and repair capacities in the retina and liver. Metabolomic analysis also demonstrated clear differences between wt and *rag1* ^−/−^ fish, as was the deficiency of the antioxidant metabolite l‐acetylcarnitine (ALCAR) in *rag1* ^−/−^ fish. Therefore, Rag1 activity does not seem to be limited to V(D)J recombination but is also involved in senescence and aging. Furthermore, we confirmed the senolytic effect of ABT‐263, a known senolytic compound and, for the first time, the potential in vivo senolytic activity of the antioxidant agent ALCAR, suggesting that this metabolite is essential to avoid premature aging.

## 1 INTRODUCTION

In mammals, recombination activating gene 1 (RAG1) and RAG2 translate a lymphoid‐specific endonuclease (RAG1/2), which initiates programmed DNA rearrangements in progenitor lymphocytes by generating double‐strand breaks (DSBs) at specific recombination signal sequences. This process, known as V(D)J recombination, assembles the vastly diverse immunoglobulin (Ig) and T‐cell receptor (TCR) genes (Bassing, Swat, & Alt, 2002).

As in other vertebrates, only one functional *rag1* locus exists in zebrafish (Willett, Cherry, & Steiner, 1997). Therefore, loss of function at this locus completely blocks Ig gene assembly and presumably removes the adaptive immune system (Wienholds, Schulte‐Merker, Walderich, & Plasterk, 2002). *Rag1* mutants (*rag1* ^−/−^) present with a point mutation that causes a premature stop codon in the *rag1* catalytic domain (Wienholds et al., 2002). In this sense, *rag1* ^−/−^ zebrafish are comparable to the *Rag1* ^−/−^ severe combined immunodeficiency (SCID) mouse model, where mutations in multiple genes (including *Rag1*) create a deficiency in the functional adaptive immune system and a loss of B and T lymphocytes (Mombaerts et al., 1992). However, similar to humans with the same condition, *Rag1* ^−/−^ SCID mice present with an increased susceptibility to infections and survive in pathogen‐free conditions only, but *rag1* ^−/−^ zebrafish reach adulthood and become fertile without obvious signs of infectious disease in standard, nonsterile aquarium facilities. In fact, it was recently reported that *rag1* ^−/−^ zebrafish respond more rapidly to a viral infection and have increased survival (García‐Valtanen et al., 2017).

However, we noticed that *rag1* ^−/−^ fish develop earlier signs of aging and present a decreased lifespan compared to wild‐type (wt) fish. This may be due to the enhanced innate immune response needed to compensate for the lack of a full adaptive immune system (Jima et al., 2009) and the associated energy cost (McDade, Georgiev, & Kuzawa, 2016), or the mutation itself may lead to premature aging due to the accumulation of DNA damage over time and the involvement of chronic inflammation and oxidative stress. On the other hand, the absence of mature lymphocytes in *rag1* ^−/−^ fish could generate a higher accumulation of senescent cells.

It was reported that the RAG family genes, in addition to their role in V(D)J recombination, may be implicated in “controlled” DNA damage for regulating gene expression, cell development, and cell fate in natural killer (NK) cells (Karo, Schatz, & Sun, 2014). Indeed, in mice, RAG‐deficient NK cells are highly cytolytic, present with an increased likelihood for apoptosis and have high levels of phosphorylated histone H2AX (γ‐H2AX), which is indicative of genomic instability; more importantly, these cells are less efficient at repairing genomic breaks during DNA damage (Karo et al., 2014).

In the present work, we used the zebrafish model and the normal development of *rag1* ^−/−^ fish in nonsterile aquarium conditions to investigate the effect of this mutation on the whole organism. The zebrafish model is emerging in biomedical research and presents many advantages, including the ability to easily maintain large stocks of fish, fast embryonic development *ex utero*, high transparency at the early developmental stages, and conserved signaling pathways. All these characteristics make it an excellent model for several human diseases (Forn‐Cuní, Varela, Pereiro, Novoa, & Figueras, 2017; Lieschke & Currie, 2007; Santoriello & Zon, 2012). The aging phenotype of zebrafish has been well described using telomerase‐deficient fish (*tert* ^−/−^), and this included sustained decrease in cell proliferation, acute apoptotic response, accumulation of DNA damage responses (DDRs), senescence, and, finally, tissue atrophy (Henriques, Carneiro, Tenente, Jacinto, & Ferreira, 2013). We assessed the premature aging in *rag1* ^−/−^ zebrafish using transcriptomic and metabolomic analyses, telomere length measurements, histological procedures, and cell cycle analysis, as well as other parameters. At the functional level, we validated *rag1* ^−/−^ zebrafish as a model of senescence by determining the effects of senolytic and antioxidant drugs on senescence regulation. Our results confirm the positive effects of senolytic drugs, such as ABT‐263 (navitoclax) and, for the first time, the potential in vivo senolytic activity of L‐acetylcarnitine (ALCAR).

## 2 RESULTS

### 2.1 *Rag1* ^−/−^ zebrafish have increased expression of immune‐related genes but decreased transcription of DNA repair genes

Using microarrays, we analyzed the transcriptome of immune‐enriched kidney marrow, which is the bone marrow equivalent in fish. A total of 1,135 genes were differently expressed in the *rag1* ^−/−^ zebrafish compared with the wt zebrafish; among these genes, 571 were overexpressed and 564 were under‐expressed (Dataset S1).

**Figure 1:**
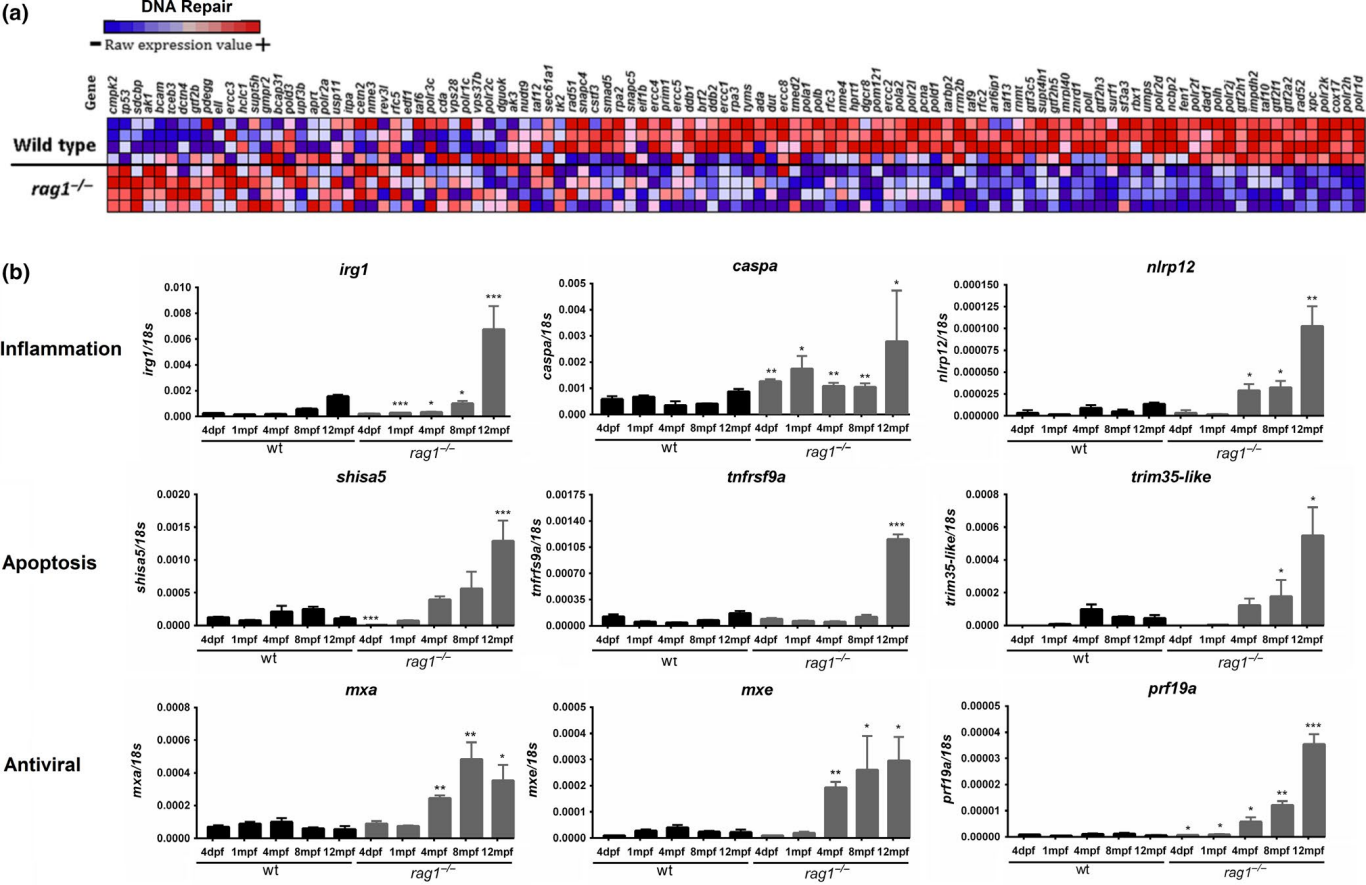
Comparative transcriptome analyses of *rag1* ^−/−^ and wild‐type zebrafish. (a) Heatmap representing the expression of the different genes included in the hallmark DNA repair. Four biological replicates from each zebrafish line (*rag1* ^−/−^ and wt) are represented in the analysis. (b) Comparison of the expression of inflammation‐, apoptosis‐, and antiviral response‐related genes in wt and *rag1* ^−/−^ zebrafish at different ages. The expression level of each gene is normalized to the expression of the *18s* gene. The graphs represent the means ± *SEM* of four independent biological replicates. Significant differences between wt and *rag1* ^−/−^ fish at each sampling point are displayed as ***(0.0001 < *p* < 0.001), **(0.001 < *p* < 0.01), or *(0.01 < *p* < 0.05)

Gene Set Enrichment Analysis (GSEA) (Subramanian et al., 2005) was used to study the processes altered in the transcriptome of the *rag1* ^−/−^ fish compared with the wt fish (Table 1). The expression of genes related to the inflammation‐, interferon alpha‐, and interferon gamma‐mediated immune responses was enriched in *rag1* ^−/−^. By contrast, genes associated with sensing DNA damage and repair were underrepresented in *rag1* ^−/−^. A heatmap showing the expression of DNA damage‐related genes revealed a decrease in the number of DNA damage response mediators and DNA repair systems (Figure 1a). For example, the genes *rad51* and *rad52*, which encode proteins that repair DSBs (Shen, Cloud, Chen, & Park, 1996), or *ddb1* and *ddb2*, which encode a complex that repairs UV‐damaged DNA (Scrima et al., 2008), were expressed at lower levels in *rag1* ^−/−^ zebrafish. By contrast, some genes, such as *tp53,* involved in DNA repair were overexpressed in *rag1* ^−/−^ zebrafish. Tp53 facilitates DNA repair, impacts the activity of various DNA repair systems, and interrupts the cell cycle to avoid the proliferation of genome‐damaged cells (Williams & Schumacher, 2016). This induction of the cell cycle arrest is reflected by the decreased transcription of genes related to cell proliferation (E2F targets, G2M checkpoint, MYC targets) in *rag1* ^−/−^ (Table 1). Other processes related to senescence, such as oxidative phosphorylation (Stöckl, Hütter, Zwerschke, & Jansen‐Dürr, 2006), unfolded protein response (Pluquet, Pourtier, & Abbadie, 2015), and fatty acid metabolism (Quijano et al., 2012), are also altered in the transcriptome of the *rag1* ^−/−^ zebrafish compared with that of the wt fish (Table 1).

**Table 1:** Gene sets from the Hallmark database were differentially enriched in *rag1* ^−/−^ and wild‐type (wt) zebrafish using Gene Set Enrichment Analysis (GSEA). In the upper part, the gene sets are enriched in *rag1* ^−/−^ compared with wt fish, and the lower gene sets are enriched in wt compared with *rag1* ^−/−^ fish

GENE SET	Normalized enriched score	*p*‐value	FDR
*rag1* ^−/−^ versus wt			
HALLMARK MYOGENESIS	1.905	0.000	0.004
HALLMARK EPITHELIAL MESENCHYMAL TRANSITION	1.611	0.003	0.096
HALLMARK IL6 JAK STAT3 SIGNALING	1.538	0.045	0.124
HALLMARK INTERFERON ALPHA RESPONSE	1.534	0.031	0.096
HALLMARK INTERFERON GAMMA RESPONSE	1.513	0.011	0.092
HALLMARK UV RESPONSE DN	1.502	0.023	0.088
HALLMARK INFLAMMATORY RESPONSE	1.487	0.020	0.084
wt versus *rag1* ^−/−^			
HALLMARK MYC TARGETS V1	−3.137	0.000	0.000
HALLMARK DNA REPAIR	−2.693	0.000	0.000
HALLMARK E2F TARGETS	−2.646	0.000	0.000
HALLMARK MYC TARGETS V2	−2.621	0.000	0.000
HALLMARK UNFOLDED PROTEIN RESPONSE	−2.506	0.000	0.000
HALLMARK GM2 CHECKPOINT	−2.254	0.000	0.000
HALLMARK MTORC1 SIGNALING	−1.883	0.000	0.002
HALLMARK UV RESPONSE UP	−1.743	0.000	0.008
HALLMARK OXIDATIVE PHOSPHORYLATION	−1.611	0.000	0.021
HALLMARK FATTY ACID METABOLISM	−1.584	0.003	0.023
HALLMARK PANCREAS BETA CELLS	−1.453	0.049	0.060
HALLMARK ESTROGEN RESPONSE LATE	−1.328	0.047	0.132

We selected genes associated with the antiviral response (*myxovirus resistance a* and *e* –*mxa* and *mxe*–, and *perforin 19a* –*prf19a*–), inflammation (*immunoresponsive gene* 1 –*irg1*–, *caspase a* –*caspa*–, and *NLR family pyrin domain containing 12* –*nlrp12*–), and apoptosis (*shisa family member 5* –*shisa5*–, *tumor necrosis factor receptor superfamily member 9* –*tnfrsf9*–, and *tripartite motif containing 35* –*trim35*–) and analyzed their expression in fish from 4 days postfertilization (dpf) until one year using quantitative PCR (qPCR) (Figure 1b). *Rag1* ^−/−^ zebrafish presented with an increase in pro‐inflammatory, apoptotic, and antiviral gene transcription over time, and the differences between the wt and *rag1* ^−/−^ zebrafish were especially evident in the one‐year‐old fish. In addition to the represented genes, microarray analysis revealed that *rag1* mutants possess a higher expression of other typical antiviral genes belonging to the repertoire of interferon‐stimulated genes (ISGs), including the *interferon‐induced 15 KDa protein* (*isg15*), different *interferon‐induced protein with tetratricopeptide repeats* (*ifit2*, *ifit5*, *ifit16*), and the teleost‐specific ISGs *grass carp reovirus (GCRV)‐induced gene 1 and 2* (*gig1* and *gig2*), among others (Dataset S1). Regarding the Toll‐like receptors (TLRs) differentially expressed in both zebrafish lines, those directly involved in the recognition of viruses (*tlr8b* and *tlr22*) were higher transcribed in *rag1* mutants (Dataset S1). Different apoptosis‐related genes such as *fas ligand* (*faslg*), *fas*, *calpain 1* (*capn1*), and *caspase recruitment domain family member 6* and *9* (*card6* and *card9*) were also affected by the mutation, as also occurs with one of the main pro‐inflammatory cytokines, the *interleukin 12ba* (*il12ba*) (Dataset S1).

Although no symptoms of infection or outbreaks were observed in *rag1* ^−/−^ zebrafish that may explain the upregulation of antiviral and pro‐inflammatory genes, bacterial load was assayed as the transcript level of 16S rRNA in wt and *rag1* ^−/−^ control samples (total visceral mass in 4‐month‐old fish) by qPCR using universal bacterial primers. No significant differences were found between both lines (Figure S1).

### 2.2 ***Rag1*** ^−/−^ **zebrafish die prematurely and show signs of senescence and aging**

**Figure 2:**
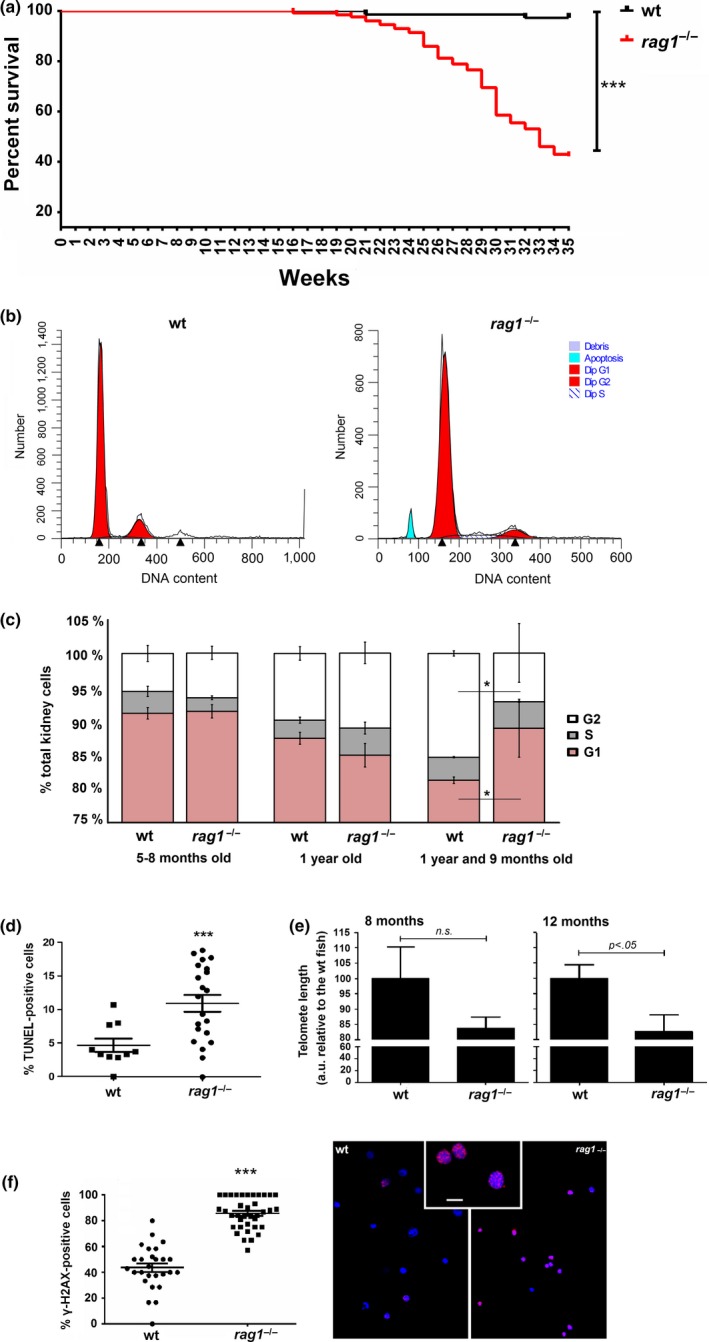
*Rag1* ^−/−^ zebrafish present with altered survival and cellular features, potentially indicating severe DNA damage. (a) Kaplan–Meier representation of the survival of *rag1* ^−/−^ zebrafish and their wt siblings (*n*  =  72 *rag1* ^−/−^, *n* =  128 wt). The log‐rank (Mantel–Cox) test was used for statistical analysis (*p* < 0.0001). (b) Cell cycle distribution of kidney cells based on the amount of DNA present. Cells were isolated from wt and *rag1* ^−/−^ fish, stained with propidium iodide, and examined by flow cytometry. Representative histograms from one wt and *rag1* ^−/−^ fish are shown. Apoptotic cells are clearly visible in the *rag1* ^−/−^ fish. (c) The percentage of cells in G0/G1, S, and G2/M was analyzed using ModFit LT 4.1 software. The stacked graphs represent the means ± *SEM* of the fish from the different groups. Significant differences in the percentage of G1 and G2/M cells were observed between wt and *rag1* ^−/−^ at the age of 1 year and 9 months (*p < 0.05). (d) Percentage of apoptotic cells in the kidneys of wt and *rag1* ^−/−^ fish, as determined by TUNEL assays. Positive cells were counted in 20 images. The means ± *SEM* are presented. (e) The dynamics of telomeres in *rag1* ^−/−^ zebrafish. Telomere length of 8‐ and 12‐month‐old zebrafish with the indicated genetic backgrounds, as assayed by FLOW‐FISH. The results are shown as the means ± *SEM* of four fish; *p* < 0.05 according to Student's *t* test. a.u.f, arbitrary units of fluorescence. (f) Immunocytochemical quantification of histone γ‐H2AX in kidney cells from wt and *rag1* ^−/−^ fish (aged 1 year and 9 months old). The number of positive cells was counted in 35 images. The means ± *SEM* are presented; *p* < 0.001 significantly different from wt fish. Confocal images of kidney cells reveal the location of histone γ‐H2AX foci in wt and *rag1* ^−/−^ fish. Nuclei are stained with DAPI and are shown as blue. Histone γ‐H2AX was observed using the Alexa Fluor 568 secondary antibody and is presented as pink. Scale bar = 25 µm. In all experiments, the results represent the means ± *SEM* of the percentage of positive cells. Mann–Whitney *U* tests were conducted to compare the means using GraphPad Prism 6 software (*p* < 0.001)

Survival analysis revealed that *rag1* ^−/−^ zebrafish die significantly early compared to their wt siblings (Figure 2a). Whereas *rag1* ^−/−^ showed an average lifespan of approximately 35 weeks, wt zebrafish survived significantly longer (*p* < 0.0001) and did not show almost mortality events after this period. At the end of the mortality experiment, most of the surviving *rag1* ^−/−^ zebrafish showed aging‐associated deformities, such as cachexia and/or lordosis, whereas these phenotypes were not observed among the wt individuals (Figure S2).

The amount of evidence that supports a link between the accumulation of DNA damage over time, senescence, and early aging is increasing (Chen, Hales, & Ozanne, 2007). One of the main phenotypic characteristics of senescent cells is permanent cell cycle arrest (Gire & Dulic, 2015). When the cell cycle was analyzed in kidney marrow cells from *rag1* ^−/−^ and wt zebrafish, we observed apoptotic cells in *rag1* ^−/−^ fish (Figure 2b) and significant differences (*p* < 0.05) between the number of G1 versus G2/M cells in both groups of fish at 1 year and 9 months of age (Figure 2c). Furthermore, increased apoptosis in kidney cells from the *rag1* ^−/−^ fish was confirmed by terminal TUNEL assay, which is a widely used technique for measuring apoptotic DNA fragmentation (Figure 2d). The percentage of apoptosis‐positive cells was twofold higher in *rag1* ^−/−^ fish than wt fish (*p* < 0.0005).

Using FLOW‐FISH, we investigated other sign of senescence, the telomere shortening. Although the telomere length of kidney cells from 8‐month‐old *rag1* ^−/−^ zebrafish was not significantly different from those of wt, telomeres were shorter in the one‐year‐old *rag1* ^−/−^ than in the wt fish (Figure 2e). Moreover, immunohistochemical analysis of basal γ‐H2AX foci, which are indicative of permanent nonrepaired DSBs (Fernandez‐Capetillo, Lee, Nussenzweig, & Nussenzweig, 2004), revealed that the percentage of γ‐H2AX‐positive kidney cells was higher in 1‐year‐old *rag1* ^−/−^ than in wt fish (Figure 2f).

**Figure 3:**
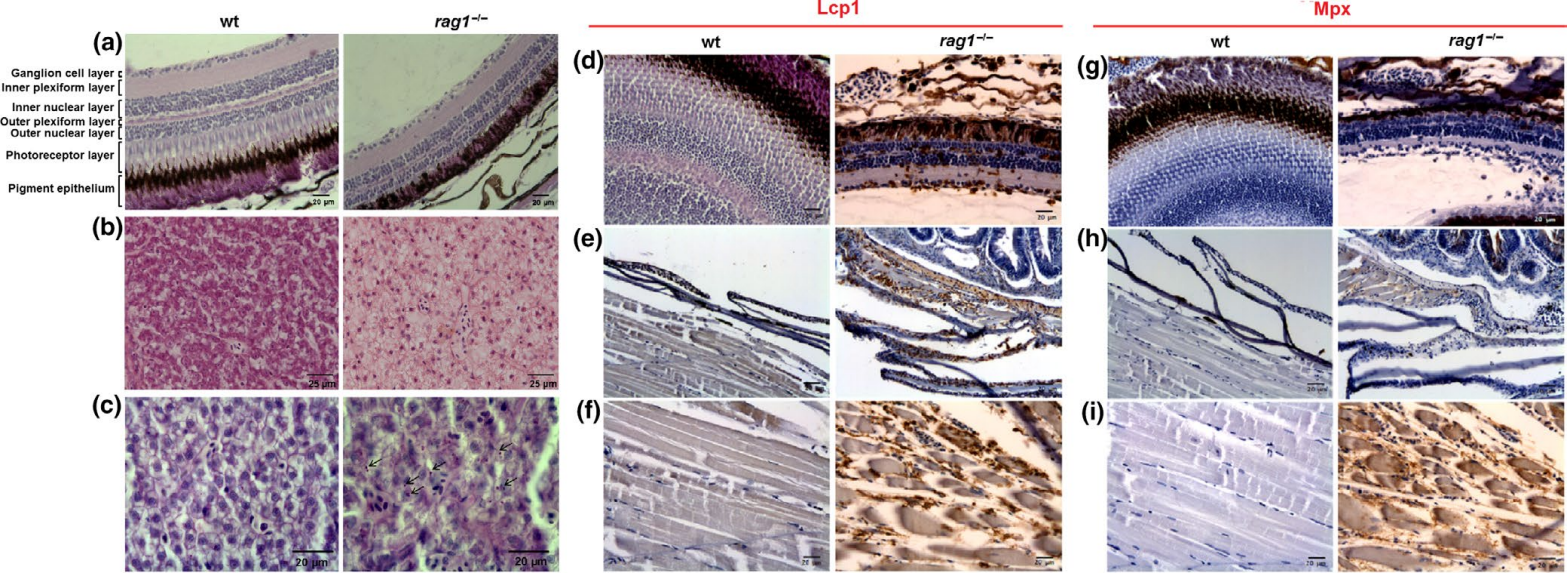
*Rag1* ^−/−^ zebrafish present with histological defects and alterations compared with wt siblings. (a–c) Premature aging signals in *rag1* ^−/−^ zebrafish. Representative images of retina (a) and liver (b, c) sections from 1‐year‐old wt and *rag1* ^−/−^ zebrafish (*n* = 5). (a) H&E‐stained eye sections revealed the loss of the photoreceptor layer and reduced thickness of nuclear layers. (b) The liver presents with hepatocyte cytoplasmic vacuolization. (c) Liver sections reveal numerous PAS‐positive areas, suggesting cytosolic accumulation of aging biomarker lipofuscin in hepatocytes (arrows). (d–i) Macrophage infiltration in *rag1* ^−/−^ zebrafish. Representative images of retina (d, g), skin (e, h), and muscle (f, i) sections from 1‐year‐old *rag1* ^−/−^ zebrafish. Consecutive anti‐Lcp‐ and anti‐Mpx‐immunostained sections reveal strong macrophage infiltration (Lcp^+^/Mpx^‐^) in the different tissues examined

Signs of aging were also detected in hematoxylin–eosin (H&E)‐stained histological eye and liver sections: The retina of 1‐year‐old *rag1* ^−/−^ fish presented without a photoreceptor layer and with reduced nuclear layer thickness (Figure 3a), while the livers presented with cytoplasmic hepatocyte vacuolization that (Figure 3b) coincided with PAS‐positive areas (Figure 3c), suggesting the cytosolic accumulation of lipofuscin, an aging, and oxidative damage biomarker (Jung, Bader, & Grune, 2007; Kishi et al., 2008).

Tissue dysfunction is usually accompanied by leukocyte infiltration. As it was previously observed, muscle and skin from uninfected *rag1* ^−/−^ zebrafish presented with significant amounts of leukocytes, whereas equivalent sections from wt fish revealed a limited leukocyte presence (García‐Valtanen et al., 2017). In this work, retina, skin, and muscle sections from 1‐year‐old *rag1* ^−/−^ zebrafish immunostained with the pan‐leukocyte marker anti‐L‐plastin (Lcp1) antibody and the neutrophil‐specific marker anti‐myeloid‐specific peroxidase (Mpx) antibody revealed a strong infiltration of leukocytes into these tissues, which resulted in negative staining for the neutrophil marker Mpx (Figure 3d–f). Therefore, these cells are macrophages (Masud et al., 2019; Mathias et al., 2009; Walters, Green, Surfus, Yoo, & Huttenlocher, 2010).

### 2.3 **Comparative metabolomics reveals oxidative stress and guanine deficiency in *rag1*** ^−/−^ **zebrafish**

**Figure 4:**
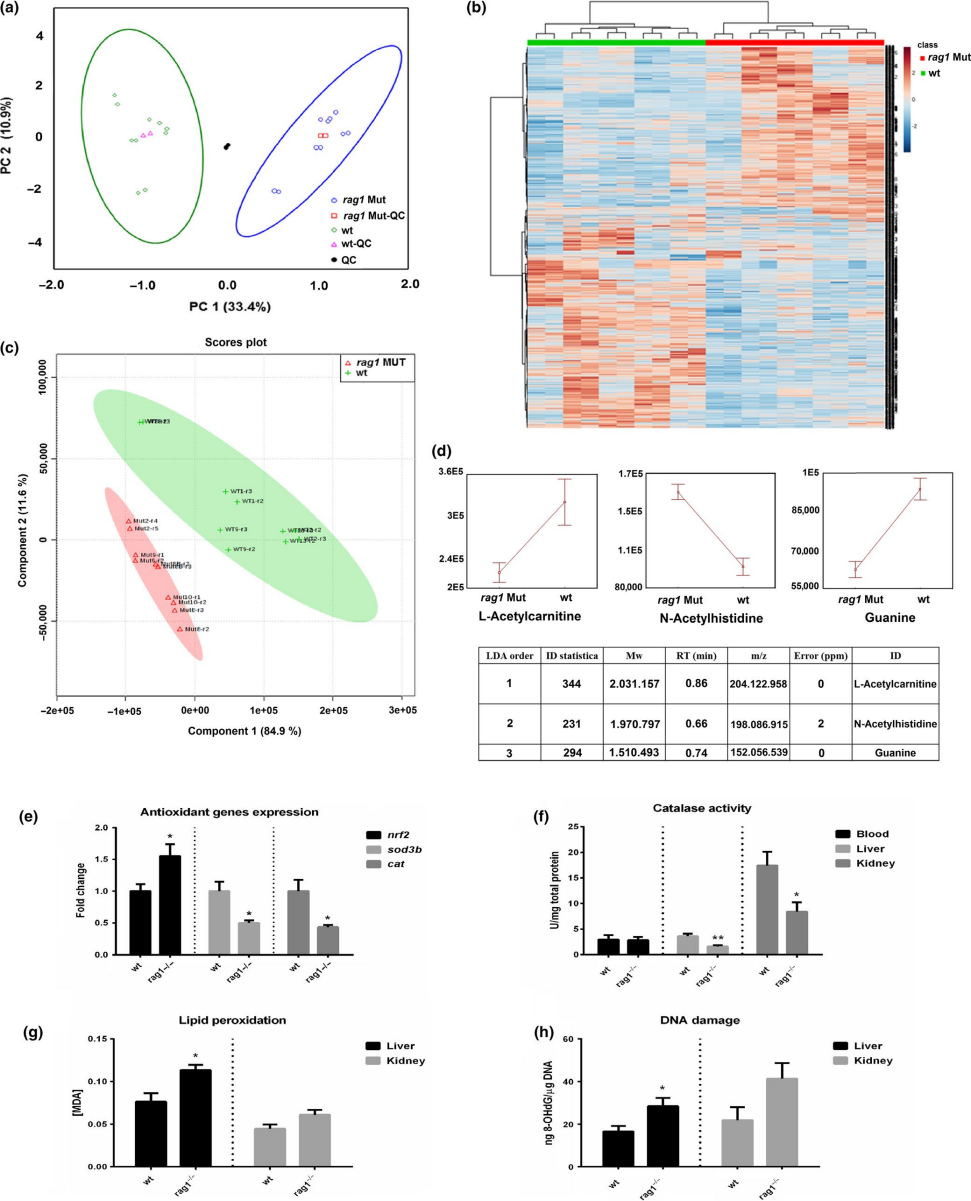
Comparative metabolomics between *rag1* ^−/−^ and wt zebrafish and analysis of their oxidative status. (a) Plot in a two‐dimensional Cartesian coordinate system, with the axes (principal components, PC) representing the greatest variations in the data from mutant (blue circles) and wild‐type (wt; green diamonds) metabolite extracts. Two injections of the *rag1* ^−/−^ quality control (QC; red squares), wt QC (pink triangles), and QC of all the samples (black circles) are represented in the plot, and 95% confidence ellipses are also included. QC samples for each group (*rag1* ^−/− ^QC, wt QC) are placed next to the middle of each cluster, assuring good data quality; in the same line, the QC (the pool of all samples) is placed near the middle of all the samples. Samples clustered into two technical replicates, indicating good analytical performance. (b) Heatmap and cluster analysis of samples with significantly different variables. (c) Score plot representing the results obtained from the PLS‐DA, with the relevant significantly different metabolites. (d) Tentative identification of metabolites included in the LDA model. Whisker plot of the mean values (± standard error, SE) of the potential metabolic markers of the mutation. The SE was calculated according to the following formula: SE = *SD*/√*n*, where *SD* is the standard deviation, and *n* is the number of observations. (e) Expression of the antioxidant genes *nrf2*, *sod3b,* and *cat* in the visceral mass of wt and *rag1* ^−/−^ zebrafish (*n* = 5). (f) Measurement of the catalase activity in blood, liver, and kidney (*n* = 7). (g) Quantification of the MDA formation as consequence of lipid peroxidation in liver and kidney from both zebrafish lines (*n* = 7). (h) Detection of 8‐OHdG in DNA isolated from liver and kidney (*n* = 4). The graphs represent the means ± *SEM*. Significant differences are displayed as **(0.001 < *p* < 0.01) or *(0.01 < *p* < 0.05)

A metabolomic study was conducted with 10‐month‐old *rag1* ^−/−^ and wt fish. A total of 16,544 signals were detected and filtered to obtain 4,620 metabolite signals with high confidence. An analysis of variance was performed to identify a total of 1,304 variables that were significantly different (*p* < 0.05) between the mutant and wt extracts. Mutant and wt samples were separately clustered, showing the high impact of mutation on the zebrafish metabolome (Figure 4a and b).

Linear discriminant analysis (LDA) was carried out with the significantly different metabolites between the groups. Three metabolites were identified as the most efficient group of variables with which to build a classification model of samples. A partial least‐squares discriminant analysis (PLS‐DA) was performed with the identified metabolites (Figure 4c), validating the potential biomarker selection. The following potential markers were identified (Figure 4d): (a) L‐acetylcarnitine (ALCAR) is an amino acid and was less abundant in the *rag1* ^−/−^ fish. ALCAR is involved in glucose and fatty acid metabolism and possesses potent antioxidant activity, reducing the oxidative stress associated with aging (Savitha, Tamilselvan, Anusuyadevi, & Panneerselvam, 2005; Singh, Mishra, & Shukla, 2016). (b) N‐acetylhistidine (NAH) levels were also higher in the *rag1* ^−/−^ fish. NAH is an unusual amino acid with a characteristic presence in the brain, retina, and lens of poikilothermic vertebrates but without a known function (Baslow & Guilfoyle, 2015). Nevertheless, NAH seems to be equivalent to the N‐acetylaspartate (NAA) metabolite in mammals (Baslow, 1997). Studies have suggested that high NAA levels are related to oxidative stress (Surendran & Bhatnagar, 2011). (c) Guanine was less abundant in the *rag1* ^−/−^ fish. This nucleotide is present in both DNA and RNA and is the most readily oxidizable of the four DNA bases, due to its low redox potential (Steenken & Jovanovic, 1997).

### 2.4 **The measurement of oxidative parameters confirms a decline of the antioxidant activity and increased oxidative cell damage in *rag1*** ^−/−^ **zebrafish**

In order to confirm the reduction of the antioxidant capacity in *rag1* ^−/−^ individuals, the gene encoding for the nuclear factor, erythroid 2 like 2 (*nrf2*) and two downstream genes encoding for the antioxidant enzymes superoxide dismutase 3, extracellular b (*sod3b*) and catalase (*cat*), were analyzed in total viscera mass from wt and *rag1* ^−/−^ zebrafish (Figure 4e). Whereas *nrf2* was higher expressed in *rag1* ^−/−^, *sod3b* and *cat* showed a lower transcription level in these fish, reflecting a decline of the antioxidant activity in *rag1* ^−/−^ compared to wt fish. Indeed, the higher transcription of *nrf2* could confirm that this decline is caused by an age‐dependent impairment of the Nrf2 signaling, as was previously reported (Zhang, Davies, & Forman, 2015). Moreover, when the catalase activity was measured in different tissues, a lower enzymatic activity in liver and kidney from *rag1* ^−/−^ fish was observed (Figure 4f).

Because this reduction of the antioxidant capacities probably leads to a higher oxidative status, lipid peroxidation and oxidative DNA damage were also analyzed in liver and kidney. Lipid peroxidation was determined through the measurement of the malondialdehyde (MDA), an end product of the polyunsaturated lipids oxidation, and DNA damage by the detection of 8‐hydroxy‐2‐deoxy guanosine (8‐OHdG), produced by the oxidation of the 2‐deoxy guanosine. A higher concentration of MDA (Figure 4g) and 8‐OHdG (Figure 4h) was found in liver from *rag1* ^−/−^ mutants.

### 2.5 ***Rag1*** ^−/−^ **zebrafish show an increased number of senescent cells, which is ameliorated by senolytic and antioxidant agents**

**Figure 5:**
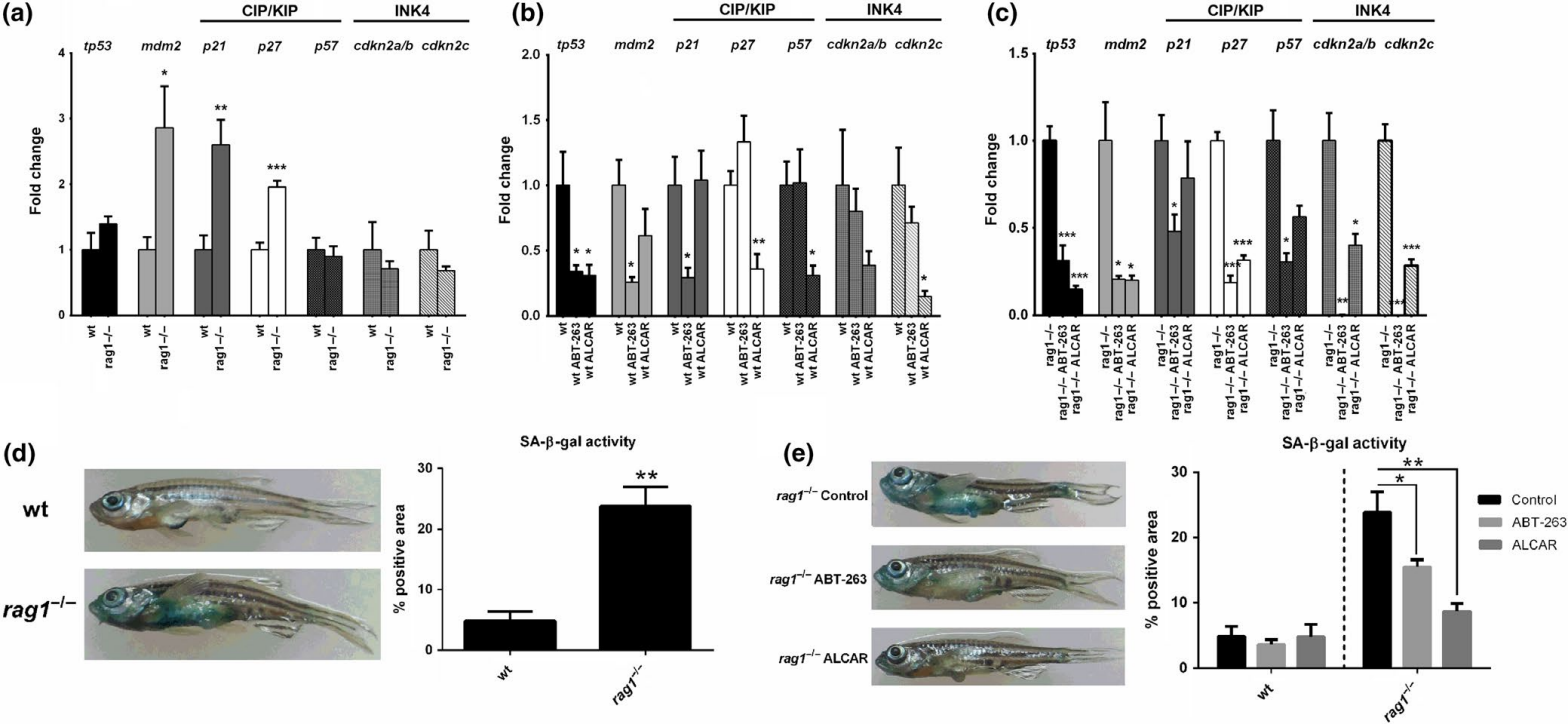
Senescence detection in *rag1* ^−/−^ zebrafish and reversion by senolytic and antioxidant treatments. (a–c) Expression of the senescence marker genes *tp53*, *mdm2*, and CIP/KIP (*p21*, *p27*, *p57*) and INK4 (*cdkn2a/b*, *cdkn2c*) inhibitors in the visceral organs of wt and *rag1* ^−/−^ zebrafish. (a) Comparison of the expression in wt and *rag1* ^−/−^ fish under naïve conditions. (b) Effect of the one‐month‐long treatment with ABT‐263 and ALCAR in wt and (c) *rag1* ^−/−^ zebrafish. The expression level of each gene was normalized to *18s* gene expression and is expressed as the fold change with respect to the levels detected in the corresponding control group (a: wt; b: wt control; c: *rag1* ^−/−^ control). (d, e) SA‐β‐gal activity in the skin of one‐year‐old zebrafish. (d) Differences in the percentage of β‐gal‐positive pixels between wt and *rag1* ^−/−^ fish under naïve conditions. (e) Effect of the one‐month‐long treatment with ABT‐263 and ALCAR in wt and *rag1* ^−/−^ zebrafish. The graphs represent the means ± *SEM* of five independent biological replicates. Significant differences are displayed as **(0.001 < *p* < 0.01) or *(0.01 < *p* < 0.05)

We conducted a functional in vivo analysis after adding the senolytic agent ABT‐263 or the antioxidant ALCAR, which is decreased in *rag1* ^−/−^ fish, to the water of 3‐month‐old wt and *rag1* ^−/−^ zebrafish. After one month, the gene expression of the senescence markers *tp53* and *mdm2*, and the inhibitors of the cyclin‐dependent kinases (CDKs) (both CIP/KIP –*p21*, *p27*, *p57*– and INK4 inhibitors –*cdkn2a/b*, *cdkn2c*–), was analyzed by qPCR, and senescence‐associated β‐galactosidase (SA‐β‐gal) activity was measured. Without treatment, the transcript levels of *mdm2*, *p21* and *p27* were higher in that *rag1* ^−/−^ fish than in their wt siblings (Figure 5a). In the wt zebrafish, ABT‐263 reduced the mRNA levels of *tp53*, *mdm2,* and *p21*, whereas ALCAR reduced those of *tp53*, *p27*, *p57,* and *cdkn2c* (Figure 5b). In *rag1* ^−/−^ fish, however, ABT‐263 affected the transcript levels of all the tested genes, while ALCAR significantly reduced the *tp53*, *mdm2*, *p27*, *cdkn2a/b,* and *cdkn2c* mRNA levels (Figure 5c). The administration of these treatments to *rag1* mutants was able to reduce the expression of the analyzed genes to lower levels than that observed in wt fish (Figure S3).

These results were further analyzed using one of the most widely used biomarkers for replicative senescence detection: Cells were histochemically stained with the artificial substrate X‐gal at pH 6.0 (Dimri et al., 1995). The external SA‐β‐gal staining analysis revealed that SA‐β‐gal activity was higher in the skin of *rag1* ^−/−^ fish than in that of wt zebrafish (Figure 5d), and the staining was reduced in *rag1* ^−/−^ fish treated with ABT‐263 or ALCAR (Figure 5e). These results indicate that *rag1* ^−/−^ fish presented with higher levels of senescent cell accumulation in the skin. The senolytic drug ABT‐263, which selectively induces apoptosis in senescent cells by inhibiting the activity of the B‐cell leukemia 2 (Bcl‐2) family of proteins (Tse et al., 2008), successfully reduced the presence of senescent cells in the skin of *rag1* ^−/−^ fish. Nevertheless, this reduction was even greater in the ALCAR‐treated fish, suggesting that oxidative stress is a factor that may induce cellular senescence in zebrafish skin.

**Figure 6:**
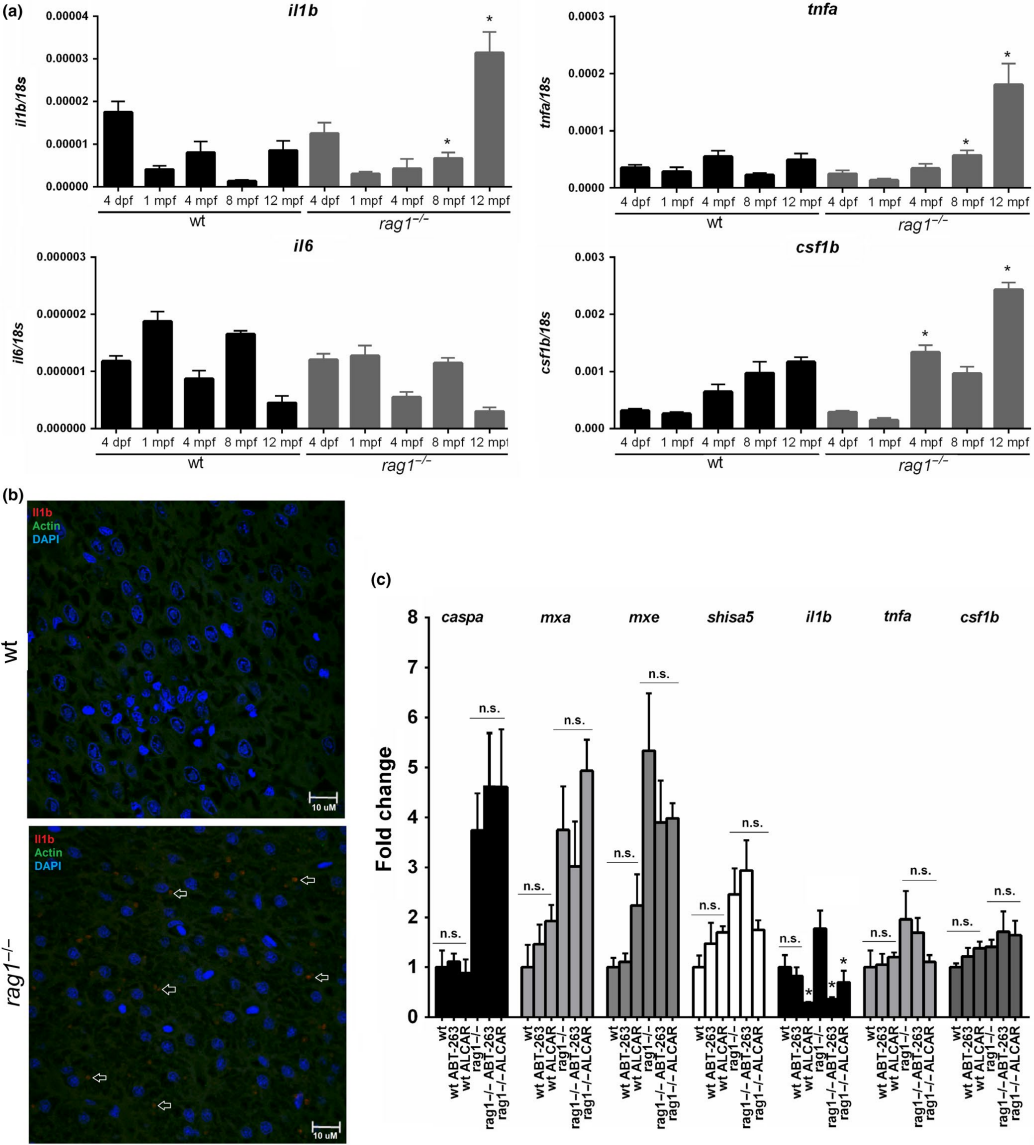
Gene expression of typical SASP components in *rag1* ^−/−^ and wt zebrafish and effect of the senolytic drugs in the expression of immune genes differentially expressed between both zebrafish lines. (a) Comparison of the expression of potential SASP genes in wt and *rag1* ^−/−^ zebrafish at different ages. The expression level of each gene is normalized to the expression of the *18s* gene. The graphs represent the means ± *SEM* of four independent biological replicates. Significant differences between wt and *rag1* ^−/−^ fish at each sampling point are displayed as *(0.01 < *p* < 0.05). (b) Il1b detection in liver sections from one‐year‐old wt and *rag1* ^−/−^ zebrafish. Arrows indicate Il1b‐positive dots. (c) Effect of the one‐month‐long treatment with ABT‐263 and ALCAR in the transcription of different genes differentially expressed between one‐year‐old *rag1* ^−/−^ and wt zebrafish. The expression level of each gene was normalized to *18s* gene expression and is expressed as the fold change with respect to the levels detected in the untreated wt fish. The graphs represent the means ± *SEM* of five independent biological replicates. Significant differences between wt and *rag1* ^−/−^ fish at each sampling point are displayed as *(0.01 < *p* < 0.05)

The expression of different senescence‐associated secretory phenotype (SASP) factors was also analyzed in wt and *rag1* ^−/−^ at different ages by qPCR. The molecules secreted by senescent cells vary depending on cell type, but some of the most commonly secreted are *interleukin 1b* (*il1b*), *tumor necrosis factor a* (*tnfa*), *interleukin 6* (*il6*), and *colony‐stimulating factor 1b* (*csf1b*). Although in the microarray analysis only *csf1b* was significantly differentially expressed between wt and *rag1* ^−/−^, qPCR revealed a higher expression of *il1b*, *tnfa,* and *csf1b* in *rag1* mutants compared to wt at the higher ages (Figure 6a). Surprisingly, the cytokine *il6* was not differentially expressed between both lines at any of the tested ages, and the lower expression values were observed for the oldest age groups (Figure 6a). Immunofluorescence detection of Il1b in different liver sections of one‐year‐old zebrafish revealed that, whereas this cytokine was almost undetectable in wt, *rag1* ^−/−^ zebrafish showed numerous Il1b‐positive hepatocytes (Figure 6b).

Next, we wanted to test the effect of ABT‐263 and ALCAR in the expression of these cytokines classically attributed to the SASP and with a higher transcription level in *rag1* ^−/−^, but also in the expression of some of the immune‐related genes checked above (*caspa*, *mxa*, *mxe,* and *shisa5*). The results showed that the higher expression of immune genes observed in *rag1* ^−/−^ zebrafish is not a consequence of the accumulation of senescent cells in these animals, and ABT‐263 and ALCAR only reduced the mRNA levels of *il1b* (Figure 6c).

## 3 DISCUSSION

While *Rag1* ^−/−^ SCID mice, as humans, present with increased infection susceptibility, *rag1* ^−/−^ zebrafish can be grown in normal aquarium conditions without mortalities (Hohn & Petrie‐Hanson, 2012; Jima et al., 2009). This ability may be related to the fact that immunity is an additive system throughout evolution, and in species with less advanced adaptive immunity, such as fish, innate immunity becomes more important (Tort, Balasch, & MacKenzie, 2003). In this work, we report an additional crucial in vivo role for Rag1 in senescence, demonstrating that the *rag1* gene mutation induces premature aging in zebrafish, which is clearly reflected in the histological retina and liver sections from one‐year‐old zebrafish and leads to a shorter lifespan.

A simple explanation for the accelerated aging observed in these mutants may be that *rag1* ^−/−^ fish pay a high maintenance cost, relying on only the innate immune response. Therefore, the increased energy resources required to fight against potential pathogens leads to faster aging. A few studies have tried to assess the energy cost of innate versus adaptive immunity, concluding that the metabolic costs of adaptive immunity are relatively low compared with those of innate immunity (McDade et al., 2016; Råberg et al., 2002). Another factor that may be linked to premature aging is inflammation: The higher innate immune activity of *rag1* ^−/−^ fish may lead to a higher inflammatory status, thereby inducing oxidative stress and reducing the antioxidant capacity (Khansari, Shakiba, & Mahmoudi, 2009).

Nevertheless, recent observations revealed an unexpected role for RAG proteins that is linked to cell fitness and DNA repair in NK cells (Karo et al., 2014). The authors propose that RAG endonuclease can generate DNA breaks outside of V(D)J recombination with the purpose of efficiently developing DNA repair mechanisms and enhancing the maintenance of the genome stability, which is required for creating long‐lived NK cells (Karo et al., 2014). If this observation is extrapolated to a whole organism, *rag1* ^−/−^ zebrafish may be accumulating DNA damage due to the absence of the RAG endonuclease activity.

On the other hand, the absence of mature CD4^+^ T lymphocytes due to the lack of adaptive immunity, and a reduced lifespan of the NK cells described by Karo et al. (2014), could be facilitating the accumulation of senescent cells in the organism. Indeed, it has been reported that these cell types, together with macrophages, play a pivotal role in the clearance of senescent cells (Hoenicke & Zender, 2012).

Therefore, three hypotheses may explain the premature senescence observed in *rag1* ^−/−^ zebrafish: (a) The absence of immune‐specific mechanisms is compensated by increased innate immune processes, implicating increased inflammation and oxygen radical production; (b) the mutation leads to DNA damage accumulation due to the defective ability of *rag1* ^−/−^ cells to successfully repair DNA breaks; and (c) the lack of mature lymphocytes and a shortened lifespan of NK cells reduce the ability of the organism to efficiently remove the senescent cells. In either case, a positive feedback loop is created in which oxidative stress and inflammation induces DNA damage, and DNA damage induces oxidative stress and inflammation. As a result, the *rag1* mutation generates DNA damage and inflammation/oxidative stress, and consequently, *rag1* ^−/−^ zebrafish present with evidence of senescence and premature aging.

Because RAG is able to interact with a broad array of places on the genome outside of antigen receptor and immunoglobulin loci, the RAG‐mediated cleavage at these sites might also generate epigenetic or gene expression modifications (Lescale & Deriano, 2017). We cannot rule out the possibility that the absence of these alternative actions of RAG in the mutant fish could be affecting their phenotype.

The *rag1* ^−/−^ zebrafish transcriptome indicated a higher expression of inflammation‐, apoptosis‐, and interferon‐related genes. Interestingly, IFN signaling activation has been previously linked with the stress response to DNA damage, and in this context, IFN activity could be related to the ability of antiviral IFNs to inhibit proliferation in cancerous cells and to promote apoptosis (Brzostek‐Racine, Gordon, Scoy, & Reich, 2011; Yu et al., 2015). Furthermore, senescent cells acquire a senescence‐associated secretory phenotype (SASP), which includes the secretion of pro‐inflammatory cytokines, including IL‐6 that promote chronic inflammation and consequently accelerate aging‐associated dysfunctions (Childs, Durik, Baker, & Deursen, 2015; Freund, Orjalo, Desprez, & Campisi, 2010). Accordingly, inflammatory and IL‐6 signaling pathways were enriched in *rag1* ^−/−^ compared to wt zebrafish. However, the transcription of the *il6* gene was not significantly different between wt and *rag1* mutants, whereas other potential SASP members were higher expressed in *rag1* ^−/−^ zebrafish. The increased accumulation of DNA damage and senescent cells in *rag1* ^−/−^ fish may induce the overexpression of immune genes. Nevertheless, the depletion of senescent cells with senolytic agents did not affect the expression of the immune genes analyzed in this work, with the exception of *il1b*. Taking this into consideration, it seems that the higher innate immunity observed in *rag1* ^−/−^ zebrafish is not a consequence of the accumulation of senescent cells. On the other hand, in the case of *il1b*, this concrete cytokine appears to be linked to the SASP.

The transcriptome analysis also revealed that the transcription of genes related to DNA repair and cell proliferation are reduced in *rag1* ^−/−^ zebrafish. The increased accumulation of DNA damage in *rag1* ^−/−^ fish is reflected in the increased percentage of γ‐H2AX‐positive cells. In response to severe DNA damage, cells undergo senescence, which implicates cell cycle arrest (Blagosklonny, 2012). The tumor suppressor *tp53* is more highly expressed in one‐year‐old *rag1* ^−/−^ zebrafish than in wt zebrafish. This gene encodes an important cell cycle checkpoint protein that regulates cell cycle progression from G1 to S phase (Smith & Pereira‐Smith, 1996; Stein & Dulic, 1995). The accumulation of irreversible DNA damage and overly short telomeres increase TP53 levels, leading to cell cycle arrest in the G0/G1 phase and generating senescent cells or favoring apoptosis (d'Adda di Fagagna et al., 2003; Kastan, Onyekwere, Sidransky, Vogelstein, & Craig, 1991; Kuerbitz, Plunkett, Walsh, & Kastan, 1992). Indeed, when the different cell cycle phases were analyzed by flow cytometry in kidney marrow cells from *rag1* ^−/−^ and wt zebrafish, the percentage of cells in G1 phase in the oldest fish was significantly higher in the *rag1* ^−/−^ fish than in the wt fish. Moreover, *rag1* ^−/−^ fish presented with an increased percentage of apoptotic cells, as determined by flow cytometry and TUNEL assays. These results are consistent with the increased caspase‐3 immunostaining in the *rag1* ^−/−^ tissues (García‐Valtanen et al., 2017).

Telomeres consist of short guanine‐rich repeats, and telomere shortening is a marker of senescence and aging (Bernadotte, Mikhelson, & Spivak, 2016; Kawanishi & Oikawa, 2004). When telomere length was analyzed in *rag1* ^−/−^ and wt zebrafish, significant telomere shortening was observed in one‐year‐old *rag1* ^−/−^ fish. Interestingly, guanine was one of the decreased metabolites in the *rag1* ^−/−^ zebrafish. With each replication, telomeres are shortened due to the inability of DNA–polymerase to work on single‐stranded 3′ ends, limiting the total number of cell divisions in somatic cells (Bernadotte et al., 2016). Oxidative stress is known to accelerate telomere shortening, especially because guanine residues are more sensitive to reactive oxygen species (ROS)‐induced DNA damage (Kawanishi & Oikawa, 2004). Indeed, a higher level of 8‐OHdG was observed in *rag1* ^−/−^ mutants. This higher oxidative status of *rag1* ^−/−^ fish was also confirmed by the measurement of lipid peroxidation.

Since general DNA damage and telomere shortening are highly induced by oxidative stress, the deficiency of the antioxidant metabolite ALCAR and the reduced activity of the antioxidant enzymes in *rag1* ^−/−^ fish may be promoting these findings. The antioxidant activity of organisms declines in an age‐dependent manner (Shigenaga, Hagen, & Ames, 1994; Tian, Cai, & Wei, 1998). Therefore, a parallel increase in ROS production and decrease in enzymatic and nonenzymatic antioxidants occurs during aging. Specifically, the content of carnitine and its derivatives, such as ALCAR, has been shown to decrease with the aging process (Costell, O'Connor, & Grisolía, 1989; Maccari, Arseni, Chiodi, Ramacci, & Angelucci, 1990), and this effect has been associated with some age‐related diseases. ALCAR supplementation has been shown to reduce oxidative stress and to ameliorate related diseases and disorders (Hagen et al., 2002; Pettegrew, Levine, & McClure, 2000).

In this work, we observed increased SA‐β‐gal activity in the skin of *rag1* ^−/−^ fish. Because aged dermis accumulates a high quantity of senescent cells (Dimri et al., 1995; Kishi et al., 2003), in zebrafish, SA‐β‐gal staining measurements have typically been conducted in the skin (Kishi et al., 2003, 2008; Tsai et al., 2007). The strong infiltration of macrophages observed in the *rag1* ^−/−^ zebrafish skin may be due to the increased accumulation of senescent cells in this tissue, since senescent cells release inflammatory chemokines and cytokines that attract leukocytes (Xue et al., 2007). We evaluated the effect of ALCAR supplementation on senescence, and the senolytic agent ABT‐263 was used as a positive control. ABT‐263 is a potent inhibitor of the Bcl‐2 family of anti‐apoptotic factors and is a promising chemotherapeutic drug for cancer cell treatment (Oltersdorf et al., 2005; Tse et al., 2008), but currently, ABT‐263 is considered a highly selective senolytic agent (Chang et al., 2016; Pan et al., 2017, 62). In addition, in vitro L‐carnitine or ALCAR administration has been proven to prevent senescent cell formation during aging due to the antioxidant activity of these compounds (Mobarak, Fathi, Farahzadi, Zarghami, & Javanmardi, 2017; Wu et al., 2014). ABT‐263 reduced *tp53*, *mdm2,* and *p21* expression in wt, but this effect was extended to *p27*, *p57*, *cdkn2a/b,* and *cdkn2c* in *rag1* ^−/−^ zebrafish. Indeed, in the mutant fish, this senolytic compound practically abolished the expression of the INK4 inhibitors. ALCAR, with the exception of *p21*, was able to significantly reduce the expression of all the analyzed genes at least in one of the lines, although, in general terms, the effect of both compounds was higher in *rag1* ^−/−^ fish. Indeed, similar to ABT‐263, ALCAR also reduced SA‐β‐gal staining in the skin, indicating that the administration of this antioxidant reduces cellular senescence. Although numerous publications have reported the positive effects of ALCAR on different age‐related parameters, to our knowledge, this is the first study to show that ALCAR reduces the number of senescent cells in vivo.

As a summary of this work, we can conclude the following: (a) the *rag1* ^−/−^ mutation accelerates aging, which is clearly supported by the numerous aging and senescence markers evaluated; (b) zebrafish are a very useful model for studying senescence in vivo*;* and (c) our study reveals that ALCAR could be considered a new biomarker for aging and its administration could reduce some aging‐related parameters, paving the way for future studies that aim to confirm the possible therapeutic use of ALCAR in premature aging syndromes.

## 4 EXPERIMENTAL PROCEDURES

### 4.1 Animals

WT and *rag1* ^−/−^ sibling zebrafish were obtained from our experimental facilities, where zebrafish are cultured following established protocols (Nusslein‐Volhard & Dahm, 2002; Westerfield, 2000). Fish care and experimental challenges were reviewed and approved by the CSIC National Committee on Bioethics under approval number ES360570202001/16/FUN01/PAT.05/tipoE/BNG. Zebrafish were sacrificed by MS‐222 overdose.

For the survival analysis, we examined *rag1* ^−/−^ (*n* = 72) and wt (*n* = 128) fish mortality during the 35 weeks (starting age 15 dpf). Kaplan–Meier survival curves were analyzed with a log‐rank (Mantel–Cox) test.

### 4.2 **Wt and *rag1*** ^−/−^ **zebrafish sampling**

To analyze the expression of selected genes during the different developmental stages of wt and *rag1* ^−/−^ zebrafish, samples were taken at 4 dpf (five biological replicates (BRs) of 10 larvae each) and at 1 (4 BRs of 4 whole fish), 4, 8, and 12 months (4 BR of 4 pooled kidney samples). Samples were stored at −80°C for RNA isolation.

Total RNA was extracted as explained below, and RNA quality was assessed with the Agilent 2,100 Bioanalyzer.

### 4.3 Transcriptome analyses

Samples from one‐year‐old wt and *rag1* ^−/−^ zebrafish were used for microarray hybridization. The 4x44K Zebrafish Gene Expression Microarray (V2, AMADID 019161) from Agilent Technologies was used to analyze differential gene expression. The data were complied with MIAME standards (Brazma et al., 2001). The signal was recorded, processed, and segmented using an Agilent G2565B scanner (Agilent Technologies) with the Agilent Feature Extraction Software (v9.5) protocol GE1‐v5_95 and an extended dynamic range, and preprocessing was completed by the Agilent Feature Extraction software v9.5.5.1.

The results for the fluorescence intensity data and quality annotations were imported into GeneSpring GX version 13.0 (Agilent Technologies), and data were analyzed as previously described (Forn‐Cuní et al., 2017). Genes differentially expressed between *rag1* ^−/−^ and wt zebrafish were identified by unpaired *t* test, and data were considered significant at *p* ≤ 0.01. The fold change cutoff was set at 2. Raw and normalized data were deposited in the NCBI's Gene Expression Omnibus (GEO, http://www.ncbi.nlm.nih.gov/geo/) and are available under the accession number GSE91397.

Microarray expression values were validated with qPCR analysis of the expression of 8 different genes in the RNA samples used to hybridize the array. qPCR and microarray fold‐changes are represented in Fig. S4, and their correlation was analyzed using Pearson's correlation coefficient.

The normalized log intensity ratios obtained were analyzed using GSEA (Subramanian et al., 2005). To characterize the differential processes enriched in the transcriptomes, the curated hallmark collection (Liberzon et al., 2015) of the Molecular Signature Database was used.

### 4.4 **Treatment of wt and *rag1*** ^−/−^ **zebrafish with ABT‐263 and ALCAR, senescence marker analysis, and SA‐β‐gal staining**

Three‐month‐old wt and *rag1* ^−/−^ zebrafish were maintained for one month with three different water treatments: ABT‐263 (2 µM + 0.1% DMSO), ALCAR (0.5 mM + 0.1% DMSO), or vehicle alone (0.1% DMSO). Tank water was refreshed every three days. After this period, twenty individuals from each group were sacrificed, and the total visceral mass was sampled, constituting 5 BR (four individuals/BR). The expression of the senescence‐related genes *p21*, *tp53,* and *mdm2* was analyzed by qPCR in these samples. The control samples were also used for analyzing the expression of *nrf2*, *sod3b,* and *cat*.

For SA‐β‐gal staining, the same treatments were conducted in one‐year‐old wt and *rag1* ^−/−^ zebrafish. After one month, fish were fixed in 2% paraformaldehyde and 0.2% glutaraldehyde in phosphate‐buffered saline (PBS) at room temperature (RT) for 30 min with agitation and then were washed 3 times in PBS (pH 7.4). Staining was conducted overnight at 37°C with agitation with the corresponding staining solution (40 mM phosphate/citrate buffer (pH 6.0), 150 mM NaCl, 5 mM potassium ferrocyanide, 5 mM potassium ferricyanide, 2 mM MgCl_2_, and 1 mg/ml X‐gal in distilled water). After 3 washes in distilled water, the whole fish was photographed in lateral view. Colorimetric quantitation of SA‐β‐gal activity staining was conducted in ImageJ software (National Institutes of Health) by calculating the percentage of the blue pixel area of each fish. The results represent the means ± *SEM* of the percentage of the blue pixel areas. Mann–Whitney U tests were conducted to compare the means using GraphPad Prism 6 (*p* < 0.001).

### 4.5 RNA isolation, cDNA transcription, and qPCR

Total RNA isolation was performed using the Maxwell 16 LEV Simply RNA Tissue Kit (Promega) according to the manufacturer's instructions. cDNA synthesis was conducted with SuperScript II Reverse Transcriptase (Invitrogen). Specific qPCR primers were designed, and amplification efficiency was calculated using seven, serial twofold dilutions of cDNA from unstimulated zebrafish with the threshold cycle (CT) slope method (Pfaffl, 2001). The primer sequences are listed in Table S1. For the analysis of the bacterial load in wt and *rag1* ^−/−^ zebrafish samples, a pair of universal primers for the 16S ribosomal RNA (rRNA) gene was used (F: 5'‐AGGATTAGATACCCTGGTAGTCCA‐3'; R: 5'‐ACTTAACCCAACATCTCACGAGAC‐3') (Campbell et al., 1995). qPCR amplifications were performed as previously described (Pereiro et al., 2015). The relative gene expression level was normalized using *18S ribosomal RNA* as a reference gene following the Pfaffl method (Pfaffl, 2001).

### 4.6 Cell cycle analysis by flow cytometry

Wt and *rag1* ^−/−^ zebrafish were divided into 3 groups according to their ages: 5‐ to 8‐month‐old, 1‐year‐old, and 1‐year‐ and 9‐month‐old animals. A total of 46 animals were individually analyzed. Kidneys were extracted and disaggregated by being passed through a 40‐µm mesh in PBS. Cells were washed twice and resuspended in PBS at 2–10 × 10^6^ cells/ml. Samples were transferred to a 15‐ml polypropylene, V‐bottomed tube and cold (−20°C) absolute ethanol was added. Cells were fixed for 3 hr at 4°C and washed twice with PBS before staining. Cells were incubated at RT, protected from light, for 40 min with a solution containing 12.5 µg/ml propidium iodide (Sigma), 200 µg/ml RNase A (Invitrogen), and 0.1% Triton X‐100 (Sigma). This incubation step ensures that the RNase has digested all the RNA, which would otherwise interfere with the DNA signal. The stained cells were analyzed using flow cytometry (FACSCalibur, Becton Dickinson) with the following settings: FL2 = 385 V; FL2‐A and FL2‐W gain 1.25; the doublet discrimination model was turned on and set to FL2; and aggregates were excluded by gating on an FL2‐width versus FL2‐area graph. ModFit LT 4.1 software was used to analyze the FL2‐A histograms and to calculate the number of cells in each step of the cell cycle (G0‐G1, S, G2‐M, and apoptotic cells).

### 4.7 **Immunocytochemical quantification of **γ**‐H2AX‐positive cells and TUNEL assays in kidney cells**

Three adult wt fish and 6 *rag1* ^−/−^ fish (1 year and 9 months old) were used in this experiment. Kidneys were sampled and individually disaggregated by being passed through a 40‐µm mesh. Cells were fixed with 2% paraformaldehyde for 15 min at 4°C and washed in PBS. Four microscope slides for each individual kidney were prepared by sample centrifugation at 800 rpm for 5 min in a Shandon Cytospin 4 cytocentrifuge (Thermo Scientific). Samples were used for immunocytochemical quantification of the histone γ‐H2AX and apoptosis quantification by TUNEL assays.

For γ‐H2AX immunocytochemistry, cells were blocked for 1 hr in PBS containing 0.1% saponin (Sigma) and 2% bovine serum albumin (BSA, Sigma) and then incubated overnight at 4°C with a rabbit polyclonal antibody against zebrafish histone H2AX (GeneTex) diluted at 1:200 in staining buffer (0.1% saponin and 0.1% BSA in PBS). The slides were washed twice and incubated with Alexa Fluor 568 goat anti‐rabbit IgG secondary antibody (Molecular Probes‐Life Technologies; 1:500) for 1 hr at RT. All slides were stained with DAPI solution (0.5 µg/ml) (Molecular Probes‐Life Technologies) for nuclear localization and mounted using ProLong antifade reagents (Life Technologies). Confocal images were captured using a TSC SPE confocal microscope and LAS AF software (Leica). The number of positive cells in the wt and *rag1* ^−/−^ fish was counted in 27 and 37 images, respectively, and the percentage of positive foci was estimated according to the number of cells per image.

The detection and quantification of apoptosis at a single‐cell level were based on DNA strand break labeling using an in situ cell death detection kit (Roche) and was analyzed by fluorescence microscopy. Briefly, cell slides were rehydrated and permeabilized for 2 min with 0.1% Triton X‐100 in 0.2% sodium citrate. After washing, the samples were incubated for 60 min at 37°C with 50 µl of TUNEL reaction mixture. A positive control sample was treated with DNase I (100 U/ml in 50 mM Tris–HCl, 1 mg/ml BSA) for 10 min at RT to induce DNA strand breaks, prior to the labeling procedures. Samples were rinsed with PBS and analyzed under a confocal fluorescent SPE microscope (Leica). The number of positive cells was counted in 20 images.

### 4.8 FLOW‐FISH

Zebrafish were euthanized, briefly rinsed in 0.5% chilled bleach, crushed, and incubated in PBS supplemented with antibiotics for 30 min. Then, the samples were centrifuged (600 *g*, 5 min), incubated in trypsin (0.5 mg/ml)/EDTA (0.1 mg/ml) in PBS for 1 min, centrifuged (600 *g*, 5 min), and then incubated in collagenase (0.5 mg/ml) in RPMI medium supplemented with CaCl_2_ 2H_2_O (0.7 mg/ml) for 30 min. The cell suspensions were obtained by pipetting, smashing, and finally filtering the digested tissues through a 100‐µm mesh and then were washed and resuspended in PBS. One million cells from each sample were then washed in 2 ml of PBS supplemented with 0.1% BSA. Each sample was divided into two replicate tubes: one pellet was resuspended in 500 µl of hybridization buffer and another in hybridization buffer without FITC‐labeled telomeric peptide nucleic acid probe as the negative control. Samples were then denatured for 10 min at 80°C under continuous shaking and hybridized for 2 hr in the dark at RT. Then, cells were washed twice in a washing solution (70% deionized formamide, 10 mM Tris, 0.1% BSA and 0.1% Tween‐20 in dH2O (pH 7.2)). The cells were then centrifuged, resuspended in 500 µl of propidium iodide solution, incubated for 2 hr at RT, stored at 4°C, and analyzed by flow cytometry within the following 48 hr.

### 4.9 Histology, immunohistochemistry, and immunofluorescence

For histological evaluation, one‐year‐old zebrafish (*n* = 5 *rag1* ^−/−^ and *n* = 5 wt fish) were euthanized, fixed in acetic zinc formalin (AZF) solution (3% zinc chloride, 5.6% formol, 1% acetic acid) for 4 days at 4°C, and decalcified in 0.25 M EDTA (pH 8) for 3 days at 4°C. Afterward, the fish were dehydrated, embedded in Paraplast Plus and sectioned at a thickness of 5 µm. Sections were stained with H&E or periodic acid‐Schiff (PAS) reagent, followed by a slight counterstain with hematoxylin. For immunohistochemistry, sections were immunostained with a 1:300 dilution of anti‐Lcp1 antibody (GeneTex) and a 1:100 dilution of anti‐Mpx antibody (GeneTex) and were slightly counterstained with hematoxylin. All sections were examined under a Leica microscope equipped with a digital camera Leica DFC 280, and photographs were processed with Leica QWin Pro software.

For zebrafish Il1b immunofluorescence, sections of 4 µm were incubated with a rabbit anti‐Il1b antibody (1:250; kindly provided by Dr. John Hansen) and a mouse anti‐actin antibody (1:500; Chemicon). The secondary antibodies used were Alexa 546 anti‐rabbit (1:500; Life Technologies) and Alexa 488 anti‐mouse (1:1,000; Life Technologies). Samples were stained with a DAPI solution (Molecular Probes‐Life Technologies) for nuclear localization. Confocal images were captured using a TSC SPE confocal microscope (Leica) using the LAS AF software (Leica).

### 4.10 Comparative metabolomics

Whole, frozen 10‐month‐old *rag1* ^−/−^ and wt zebrafish (*n* = 5/group) were ground in liquid nitrogen using a mortar and pestle, and the fish powder was weighed. A mixture of 80:20 methanol:water was used as the extraction solvent at −80°C. Samples were mixed and allowed to rest for 15 min at 4°C, and then, the supernatant was clarified by centrifugation at 13,000 *g* for 10 min, removed, and stored at −80°C for LC‐MS/MS analysis. Metabolomic analyses were performed using an UHPLC system 1,290 (Agilent Technologies) connected to an Agilent 6,540 quadrupole‐time‐of‐flight mass spectrometer (Q/TOF MS) equipped with an orthogonal ESI interface (Agilent Jet Stream) and operating in positive ion mode. The instrument was controlled by a PC running Mass Hunter Workstation software 4.0 from Agilent. The sample was injected into an Agilent ZORBAX C8 Rapid Resolution HD system and maintained at 40°C. Each sample was analyzed in triplicate. Reverse‐phase chromatographic separation was performed using water with 0.1% formic acid as phase A and acetonitrile with 0.1% formic acid as phase B. Accurate TOF MS mass spectra were recorded across the range of 50–1000 m/z at 1.5 spectra/s. After all samples were analyzed in duplicate, raw LC‐MS data were processed using R language. Signal detection, deconvolution, and alignment were performed using XCMS and mzMatch packages. Then, a recursive analysis was carried out using the PeakML.Gapfiller tool. Parameters for data processing included the following: peak detection tolerance = 40 (ppm); peak width = 5–100 (s); S/N ratio = 3; alignment tolerance = 20 (ppm); and recursive analysis tolerance 40 (ppm). Potential metabolite markers were tentatively identified by matching the obtained m/z values to those published in free‐access databases, including the KEGG (Kanehisa et al., 2008), HMDB (Wishart et al., 2009), and METLIN database (Smith et al., 2005).

### 4.11 Measurement of the catalase activity, lipid peroxidation, and oxidative DNA damage

The three experiments were conducted in 1‐year‐old zebrafish and by following the manufacturer's recommendations. For catalase activity evaluation blood, liver and kidney were sampled (*n* = 7) and the Catalase Colorimetric Activity Kit (Invitrogen) was used. The enzymatic activity was referred to the total protein content of each sample. The experiment was replicated three times. Lipid oxidation was analyzed in liver and kidney (*n* = 7) with the Lipid Peroxidation (MDA) Assay Kit (Sigma). This experiment was replicated twice. Finally, the detection of 8‐OHdG was conducted in DNA extracted from liver and kidney (*n* = 4) with the DNA Damage (8‐OHdG) ELISA Kit (StressMarq Biosciences). In this case, the concentration of 8‐OHdG was referred to the DNA contain of each sample.

## CONFLICT OF INTEREST

None declared.

## AUTHOR CONTRIBUTIONS

BN, AE, and AF have conceived and designed this work. MV, GF‐C, *SD*, JC, and AF conducted the transcriptome analyses. MV and AR carried out the cell cycle analysis by flow cytometry and TUNEL assay. Immunocytochemical quantification of γ‐H2AX‐positive cells was conducted by *SD* and AR. MA and MLC analyzed the telomere length. AL‐M, VM, and *SD* carried out the histology and immunohistochemistry. AM‐L, RM‐G, and AE were implicated in the metabolomics analysis. PP performed the experiments with ABT‐263 and ALCAR, senescence marker analysis, and the SA‐β‐gal staining together with MC. BN, PP, and AF wrote the manuscript.
