# Supplementary material for: Rag1 immunodeficiency‐induced early aging and senescence in zebrafish are dependent on chronic inflammation and oxidative stress
Source: Aging Cell. 2019 Jul 26;18(5):e13020. doi: 10.1111/acel.13020 (PMC6718522; doi:10.1111/acel.13020)

Figure S1. Measurement of bacterial load in wt and *rag1*$^{-/-}$ zebrafish visceral mass by qPCR analysis of the 16S rRNA gene using a pair of universal primers. The expression level of the 16S rRNA gene is normalized to the expression of the zebrafish 18s rRNA gene. The graphs represent the means ± SEM of 5 independent biological replicates.

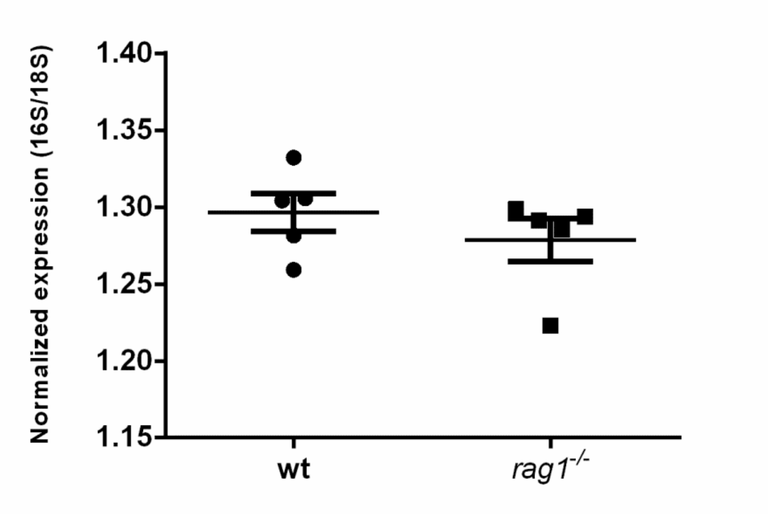

Supplement: Supplementary file 1 [file ACEL-18-e13020-s001.pdf]
