# Supplementary figures and images for: Rag1 immunodeficiency‐induced early aging and senescence in zebrafish are dependent on chronic inflammation and oxidative stress
Source: Aging Cell. 2019 Jul 26;18(5):e13020. doi: 10.1111/acel.13020 (PMC6718522; doi:10.1111/acel.13020)

**Figure S2. Phenotype of 35-weeks-old wt and *rag1*<sup>-/-</sup> zebrafish.**

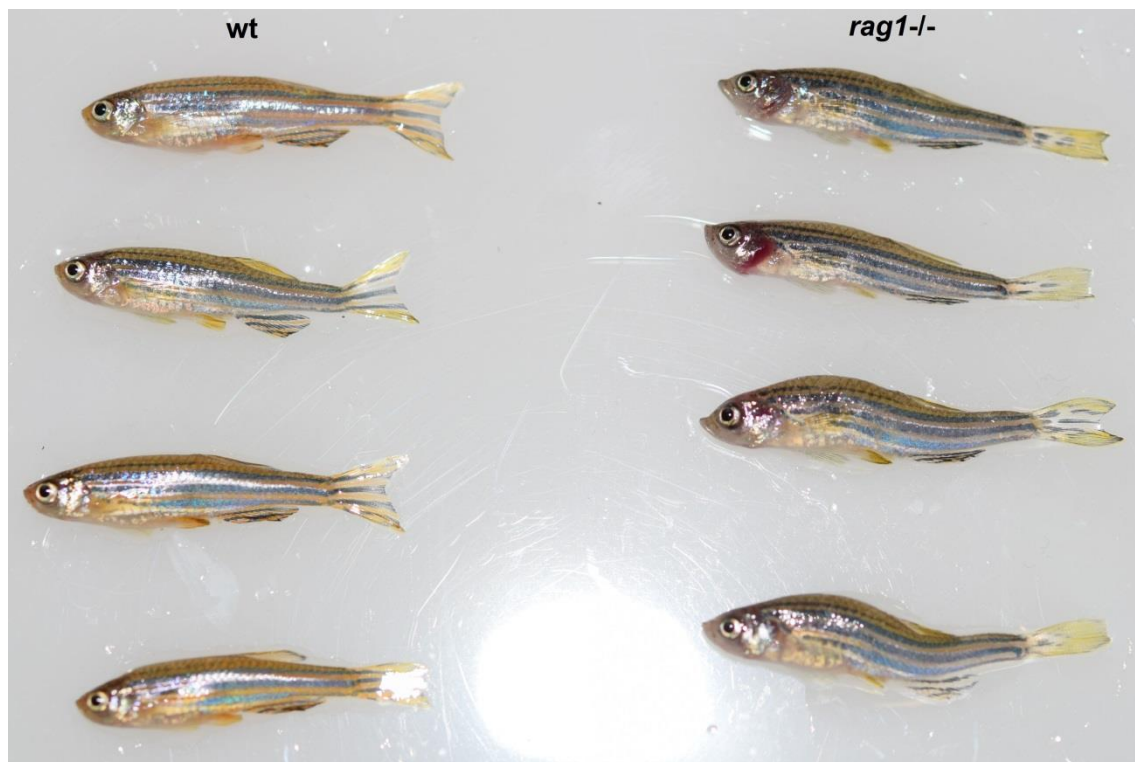

Supplement: Supplementary file 2 [file ACEL-18-e13020-s002.pdf]
