# Supplementary material for: Rag1 immunodeficiency‐induced early aging and senescence in zebrafish are dependent on chronic inflammation and oxidative stress
Source: Aging Cell. 2019 Jul 26;18(5):e13020. doi: 10.1111/acel.13020 (PMC6718522; doi:10.1111/acel.13020)

Figure S3. Effect of ABT-263 and ALCAR in the mRNA expression of different senescence and cell cycle-related genes.

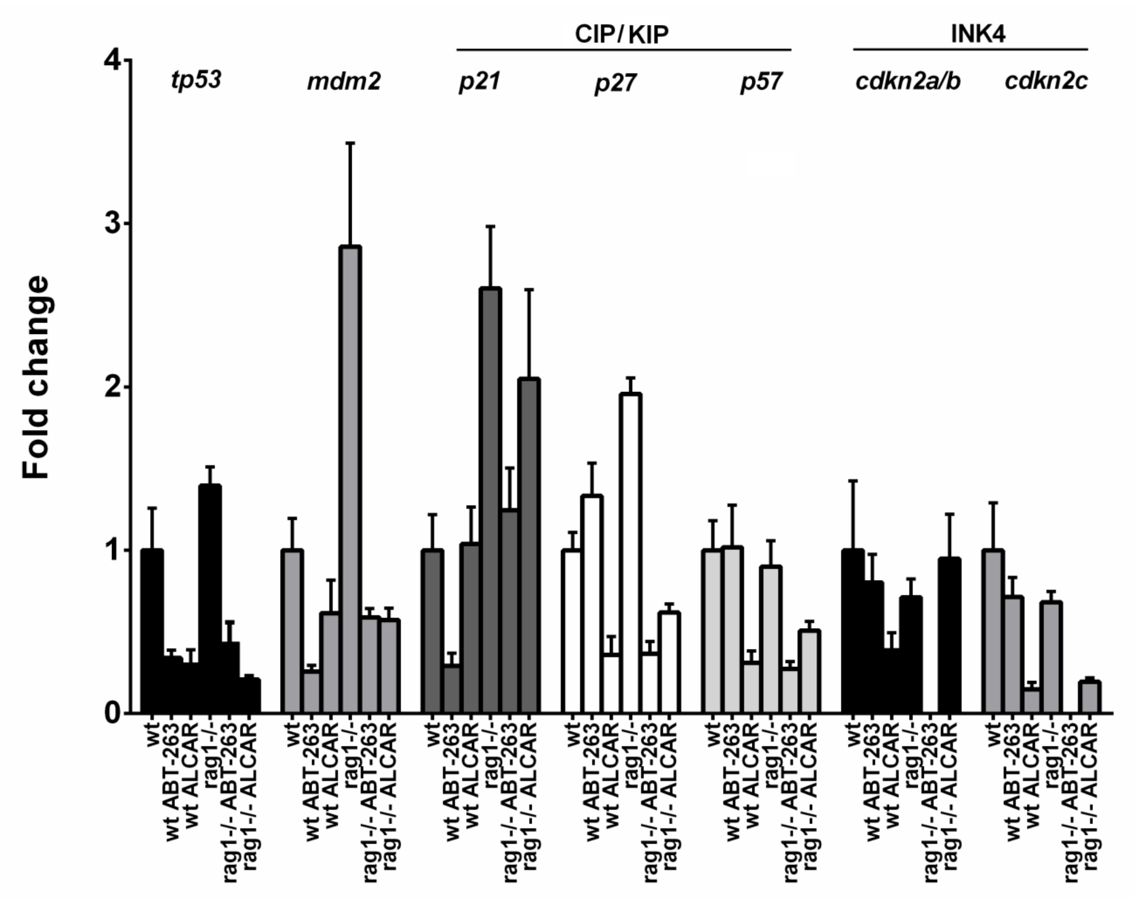

Supplement: Supplementary file 3 [file ACEL-18-e13020-s003.pdf]
