# Supplementary material for: Rag1 immunodeficiency‐induced early aging and senescence in zebrafish are dependent on chronic inflammation and oxidative stress
Source: Aging Cell. 2019 Jul 26;18(5):e13020. doi: 10.1111/acel.13020 (PMC6718522; doi:10.1111/acel.13020)

Figure S4. Microarray validation. A group of eight candidate genes were selected for quantifying their expression pattern by qPCR and validate the microarray analysis. The correlation between both data was analyzed using the Pearson´s correlation coefficient (r=0.963; p<0.001).

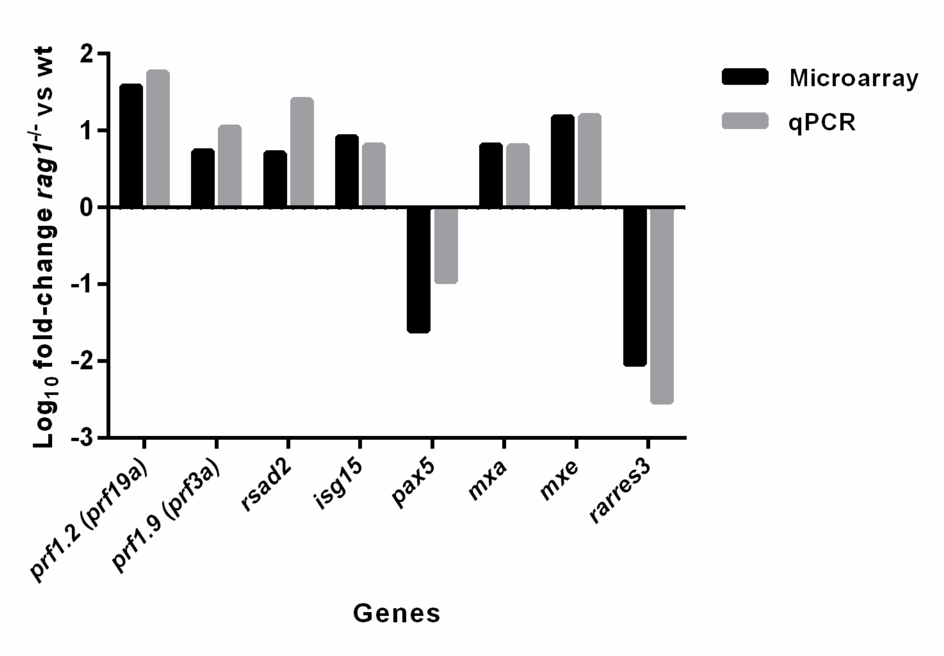

Supplement: Supplementary file 4 [file ACEL-18-e13020-s004.pdf]
