# Supplementary material for: Rag1 immunodeficiency‐induced early aging and senescence in zebrafish are dependent on chronic inflammation and oxidative stress
Source: Aging Cell. 2019 Jul 26;18(5):e13020. doi: 10.1111/acel.13020 (PMC6718522; doi:10.1111/acel.13020)
Supplement: Supplementary file 6 [file ACEL-18-e13020-s006.pdf]

Table S1. Primer pairs used in this study.

Gene	Ensembl ID	Sense	Sequence (5'→3')
shisa5	ENSDART00000105846	F	TGCAGGATGTGCAGAAGAAC
		R	GGTGGATATGGTCCCACTTG
tnfrsf9a	ENSDART00000150153	F	TGTGGATATGACCCAGCAGA
		R	ATCTCCACACCGAAATCCAG
trim35-like	ENSDART00000082383	F	AATCACAAGTGCTCCCCTGT
		R	TTTCAGCCTGTTCCATGCAG
irg1	ENSDART00000101982	F	CAACGTTCAGTCCTTCAGCA
		R	GTTTCCTCCAGTGACCGTAA
caspa	ENSDART00000034544	F	AAAAGGAGCGGCTCAGAGAA
		R	CACCCATAATGGCGTCTCTT
nlrp12	ENSDART00000138617	F	TCCAGTCTCTCGGCTTGAAA
		R	GCATTCTCACTGCTCCATCA
mxa	ENSDART00000122566	F	CACAGACAATCATGCCACCT
		R	TTTGCAGCTCCAAAGCAGCT
mxe	ENSDART00000110445	F	AGTCACCCAATGTCAGTGCA
		R	GCTGAGAGATGTACTGGTTC
prf1.2 (prf19a)	ENSDART00000133277	F	TGAGGCACGTACCAGTTTAC
		R	TCACGTAAGTACCTTTGCGC
prf1.9 (prf3a)	ENSDART00000085736	F	GGTTCCTGTAGCTCTACTGT
		R	GCTTCTGCTTTTCGTTCTGC
rsad2	ENSDART00000019617	F	GCCGAGTGTCAGCATTGTTA
		R	CCTGTGCTCGACCTATAAGT
isg15	ENSDART00000130554	F	AGAAGGGCCAGGTCAAAACT
		R	CGAGCTGTCTGCCTTTGAAA
pax5	ENSDART00000054395	F	GCAGAGAGAGTGTGTGACAA
		R	TGCGTCACTGCCACGGTAGA
rarres3	ENSDART00000110276	F	TCCTTACGTGGCTCATCTGA
		R	ATGCTTGTCGTCCAGGTAGT
nrf2	ENSDART00000062854	F	TGTCACTCCCAGAGTTGCAG
		R	CACACTTCTGTTTGAGCCGA
sod3b	ENSDART00000112150	F	TCAGTTGCCATGTGCTCTTC
		R	TCACGCTCAGCTTTTCCTTT
cat	ENSDART00000166496	F	CGCTTCTGTTTCCGTCTTTC
		R	GGAATCCCTCGATCACTGAA
tp53	ENSDART00000177458	F	GATGGTGAAGGACGAAGGAA
		R	AAATGACCCCTGTGACAAGC
mdm2	ENSDART00000077854	F	AACTCCCAACACAACCTTCG
		R	GGCTGTGATGATGTGGTTTG

p21	ENSDART00000136722	F<br>R	AACGCTGCTACGAGACGAAT<br>CGCAAACAGACCAACATCAC
p27	ENSDARG00000054271	F<br>R	TGAAGCCTGGAACTTCGACT<br>TGTGAATATCGGAGCCCTTC
p57	ENSDARG00000010878	F<br>R	TGAGATGAAACGCAAACTGC<br>CCTCCCACTCGTAATCTCCA
cdkn2a/b	ENSDARG00000037262	F<br>R	TGAACGTCGAGGATGAACTG<br>AAGGTGCGTTACCCATCATC
cdkn2c	ENSDARG00000057610	F<br>R	TGGATGTCGTGCAGTTTCTC<br>ATGCTCTCCAACCACTCCAC
il1b	ENSDARG00000098700	F<br>R	TTCCCCAAGTGCTGCTTATT<br>AAGTTAAAACCGCTGTGGTCA
tnfa	ENSDARG00000009511	F<br>R	ACCAGGCCTTTTCTTCAGGT<br>GCATGGCTCATAAGCACTTGTT
il6	ENSDARG00000102318	F<br>R	TCAACTTCTCCAGCGTGATG<br>TCTTTCCCTCTTTTCCTCCTG
csf1b	ENSDARG00000068263	F<br>R	GCATTCGAGACGCAATACAA<br>GTACTCTTGCTCGCAGGTCC
18s	ENSDART00000126982	F<br>R	ACCACCCACAGAATCGAGAAA<br>GCCTGCGGCTTAATTTGACT
